# A qualitative study of undergraduate clerkships in the intensive care unit: It’s a brand new world

**DOI:** 10.1007/s40037-017-0349-x

**Published:** 2017-04-07

**Authors:** Enda O’Connor, Michael Moore, Walter Cullen, Peter Cantillon

**Affiliations:** 1 Department of Anaesthesia and Intensive Care Medicine, St. James’s Hospital and Trinity College, Dublin, Ireland; 2 Department of Anaesthesia and Intensive Care Medicine, St. James’s Hospital, Dublin, Ireland; 3 Department of Urban Primary Care, University College Dublin, Dublin, Ireland; 4 Department of Primary Care, National University of Ireland, Galway, Ireland

**Keywords:** Qualitative research, Clinical workplace learning, Undergraduate education, Intensive care medicine, Social cognitive theory

## Abstract

**Introduction:**

While ICU clerkships are commonplace in undergraduate medical education, little is known about how students learn there. This study aimed to explore students’ perceptions of the ICU as a learning environment, the factors influencing their learning and any perceived differences between learning in the ICU and non-ICU settings.

**Methods:**

We used interpretivist methodology, a social cognitive theoretical framework and a qualitative descriptive strategy. Ten medical students and four graduate doctors participated in four semi-structured focus group discussions. Data were analyzed by six-step thematic data analysis. Peer debriefing, audit trail and a reflexive diary were used.

**Results:**

Social cognitive influences on learning were apparent in the discussions. Numerous differences emerged between ICU and non-ICU clinical clerkships, in particular an unfamiliarity with the environment and the complex illness, and difficulty preparing for the clerkship. A key emergent theme was the concept of three phases of student learning, termed pre-clerkship, early clerkship and learning throughout the clerkship. A social cognitive perspective identified changes in learner agency, self-regulatory activities and reciprocal determinism through these phases. The findings were used to construct a workplace model of undergraduate intensive care learning, providing a chronological perspective on the clerkship experience.

**Conclusions:**

The ICU, a rich, social learning environment, is different in many respects to other hospital settings. Students navigate through three phases of an ICU clerkship, each with its own attendant emotional, educational and social challenges and with different dynamics between learner and environment. This chronological perspective may facilitate undergraduate educational design in the ICU.

## What this paper adds

Undergraduate intensive care clerkships are common. Qualitative research about student ICU learning is lacking. The ICU is a more challenging learning environment than non-ICU settings. The ICU rewards students with numerous positive learning opportunities. A chronological workplace model with a social cognitive framework can be used to better understand student learning during ICU clerkships. This model could be applied to guide ICU educational design and may find application for clinical workplace learning in other hospital settings.

## Introduction

Intensive care medicine (ICM) is a specialty that is becoming a standard discipline in undergraduate medical curricula [1, 2]. An ICM clinical clerkship offers several potential benefits when compared with those in a non-intensive care setting. Its ‘horizontal’ structure enables simultaneous exposure to numerous subspecialties [2]. It facilitates vertical integration between basic and clinical sciences and exposes students to the clinical and procedural aspects of acute and critical illness, infrequently found elsewhere [3, 4].

The intensive care unit (ICU), however, with its unique customs and norms, may be a challenging learning environment for students. They may feel intimidated by the unfamiliarity of the surroundings and the complexity of pathology and treatments [5], promoting uncertainty and disengagement, thereby hindering learning [6, 7]. Unfamiliar departmental culture and team structures may interfere with early clerkship socialization and identity formation [7]. Moreover, active student participation, important for effective learning [8–11], may be harder to achieve in the complex, high-stakes ICU setting.

Numerous qualitative studies have evaluated undergraduate clinical workplace learning [7, 8, 11–16] but none of these studies includes data on ICM clerkships. Existing research about undergraduate ICM learning, mostly quantitative with self-selected students doing 2‑ or 3‑month electives, suggests that it is popular and achieves measurable learning outcomes [17–22]. To date, therefore, analysis of medical student learning within an ICU from an interpretive viewpoint is lacking.

A key aspect of clinical workplace learning is the interaction between the environment and the individual [23]. Social cognitive theory (SCT) views learning as the consequence of an individual’s interpretation of and interaction with an environment. It identifies with learners as ‘agents of experiences rather than simply undergoers of experiences’ (p4) [24]. The learner, through her thoughts, actions, motivations and behaviour – learner agency – exerts an influence over the learning environment. This dynamic process, whereby the environment, learner and behaviour all exert mutual determinative effects on learning, is known as reciprocal determinism [25, 26]. Central to this theory is the concept that an environment, for example an ICU, is a potential learning space until a process of ‘actualization’ is initiated by the arrival therein of an active, cognitive learner [25, 27]. How or whether this process occurs is in turn influenced by several factors, in particular the type of social environment, a learner’s emotional state and their skills in self-regulation – identifying learning goals and adopting strategies to achieve these goals [24, 28, 29].

SCT may be a suitable lens through which to view medical student learning in the ICU. The challenging nature of the environment may hinder the student’s ability to manipulate and self-regulate their learning or to exert a determinative influence there. Accordingly, they may not progress from observers of learning to agents of learning, the ICU may remain a potential learning space and the benefits of the ICU as an educational experience may not be fully realized. The suitability of SCT is further strengthened by its prior use in the evaluation of medical workplace learning, though in a non-ICU setting [14, 30].

Therefore, although much is known about how students learn in the clinical setting, student experience in the ICU is not represented in the current published literature. Qualitative research might offer valuable insights into these experiences. With an overarching motive to optimize undergraduate intensive care learning in the study institution, the study objective was to address this gap in current knowledge. Using an SCT framework, an interpretivist methodology and a qualitative descriptive strategy [31, 32], the purpose of this study was to answer the following research questions:

- how do students describe the learning experience of an ICM clinical clerkship? and
- what are the factors that hinder and help learning during the ICM clerkship?

## Methods

### Context

The study was carried out at the Faculty of Health Sciences, Trinity College, Dublin, Ireland. The 5‑year medical curriculum comprises 2 years of pre-clinical basic sciences and 3 years of clinical skills training with clerkships in hospital and community settings. All students undergo an ICM clerkship in either year 3 or 5 in one of two tertiary-level ICUs. This is a 2-week placement during which students actively participate in all clinical and educational team activities, including bedside ward rounds, multidisciplinary case discussions, ICU procedures, family discussions and scheduled junior doctor teaching.

### Study design

Following ethics approval from the Faculty of Health Sciences Research and Ethics Committee, study participants were selected by purposive sampling [33, 34] from a sampling frame of 3rd and 5th year medical students, and 1st year medical graduates (interns) who had a prior clinical rotation in ICM. Invitations to participate, and recruitment, were conducted by a third party. Informed consent was obtained from all participants. Sample size was guided by principles of data saturation [35].

Data were collected using focus group discussions [36, 37]. Each of the three target groups – 3rd/5th year students, and graduate doctors – was allocated to separate discussions, thereby enabling source triangulation [38]. In total, 14 participants (10 students and 4 graduate doctors) took part in four focus groups (2–4 participants per group; 50–73 minutes duration) between March–May 2015. There was an equal gender distribution. Participants had undergraduate experience in seven different ICUs in Ireland, either during the 3rd year (*n* = 6) or 5th year (*n* = 8) of medical school. A pilot focus group was conducted with two final year students and the data collected included in final data analysis [39].

To minimize moderator bias and to enhance truthfulness, student focus groups were moderated by a 1st year graduate doctor who had no clinical duties in the ICU (MM) with the principal researcher (EOC) as observer [40]. The graduate focus group was moderated by EOC. All focus groups were audio recorded and transcribed verbatim by an independent third party. To optimize accuracy, each transcript was reviewed for errors, and study participants were invited to member-check the transcripts [41]. No revisions were requested.

A semi-structured question format was adopted, to balance the requirement for a theory-driven deductive enquiry with the capacity to discover unanticipated, emergent data [42]. Structured questions explored concepts related to reciprocal determinism and to factors influencing the active learner in the ICU. The remaining questions sought general comments about the clerkships to stimulate unstructured enquiry. The questions were piloted as part of the study design (see Table 1 for topic guide).

**Table 1:** Question topic guide for the focus group discussions

1)	Do you think the rotation in the intensive care unit was a positive or negative experience for you?
2)	If you think about when you were walking around with the ICU team, could you make sense of what you were seeing and hearing?
3)	How did you feel going into the ICU for the first time?
4)	The next thing I’d like you to think about is; during the rotation, did you feel like an insider (a member of the team), or an outsider (an observer)?
5)	Thinking back to what we’re discussed so far, do you think that the ICU rotation was different to other clinical rotations you have done?
6)	We have had students who, after attending 2–3 days of their ICU rotation, have not appeared for the rest of the 2 weeks. Is there anything about the ICU rotation that would make a student less likely to attend?
7)	Do any of you have anything else to add that you think is relevant to these dicussions?

Coding was performed by the principal researcher (EOC) using thematic analysis, following a six-step strategy proposed by Creswell [43]. Data analysis software (nVivo 10; QSR International 2012) was used. Rather than coding pre-ordinately, the ‘codes themselves derive(d) from the data responsively’ (p560) [42]. The principal researcher (EOC) maintained a reflexive diary, articulating his positionality and potential sources of researcher bias, in addition to strategies to minimize their influence on data collection and analysis [41]. Subsequent peer debriefing with a senior co-researcher (WC) did not result in any changes to the analysis, interpretation or final report of the data.

Acknowledging the risk of bias in this insider research study as well as the principal researcher’s motivation to improve student learning, the validity of the study findings was enhanced by several methods. In addition to data triangulation and peer debriefing, an audit trail, using screenshots from analysis software during coding, mapped the stepwise process of data analysis [33].

## Results

A majority of participants described the learning experience as *‘positive’, ‘a nice experience’, ‘enjoyable’ *or *‘really good’,* sometimes with qualifications (*‘It was very good but I also found it very tough emotionally’ *[FG3;F2]).

A key emergent theme was how the students’ physical, emotional and social engagement with the clerkship underwent a process of change, much of which could be explained in terms of SCT, characterized by negative emotions, apprehension and uncertainty prior to the clerkship, moving through a period of orientation and increasing familiarization, before reaching a state where positive learning experiences could occur. We divided this process into three stages which we termed (1) pre-clerkship, (2) early clerkship and (3) learning throughout the clerkship. Subthemes relevant to each of these phases emerged in the discussions (Table 2). Furthermore, a majority of these subthemes related to the differences between ICU and non-ICU clerkships.

**Table 2:** Student learning in intensive care medicine: the three phases of the clerkship and associated sub-themes

Clerkship phase	Associated sub-themes
1) Pre-clerkship	Perceived lack of familiarity/ complexity and severity of illness* Perceived uselessness during clerkship* Difficulty preparing for the clerkship* Personality of the student
2) Early clerkship	Initial interaction with ICU staff* Students’ sense of belonging Structure/organization of the clerkship* Level of guidance available*
3) Learning throughout the clerkship	Involvement in patient care* Adjustment in learning method* Value of observing and experiencing* Reciprocal dynamics between student and learning environment Students’ self-learning activities(personal agency)

*Subthemes for which there was a reported difference between ICU and non-ICU clerkships

### Stage 1: Pre-clerkship

Numerous terms were used to describe the anticipation of the ICM clerkship: *‘scary’, ‘terrifying’, ‘intimidating’, ‘nerve-wracking’*. A variety of negative emotions was reported including* ‘very apprehensive’ *and* ‘anxious’. *Four subthemes in this stage of learning pertained to hurdles facing the novice learner anticipating an unfamiliar clinical environment, and to factors influencing self-regulatory strategies such as setting learning goals and being emotionally and cognitively prepared for the clerkship.

#### Lack of familiarity/Complexity and severity of illness

Participants reported unfamiliarity with both non-medical and medical aspects of the ICU. The ICU was described as; *‘it’s a brand new world’* (FG2;M2) and *‘… one of those closed areas in the hospital that you’ve never been before’* (FG4;M3). The new environment contains potential hazards for novice learners, such as *‘I don’t know what I can touch, … I don’t want to touch anything in case I unplug something’ *(FG2;F2) and* ‘… even the technology and stuff … you wouldn’t know how to use them’ *(FG2;F1)* and ‘… the things that made me most apprehensive … when I don’t know where I’m going …’ *(FG3;F1).

The medical unfamiliarity pertained to students’ prior inexperience of severe, complex illness: *‘… there was like an edge of discomfort, … apprehension at how sick the patients might have been’ *(FG3;M2) and* ‘… seeing patients very sick, in the ICU that would be very terrifying’ *(FG2;M2).

#### Perceived uselessness during the clerkship

There was a strong pre-clerkship perception that students are not ‘useful’ in the ICU. This sentiment was captured in several statements; ‘*there’s effectively nothing we can do’ *(FG2;F1)*; ‘I wasn’t of any use really’ *(FG3;M1). This was balanced against students’ urge to have a role to play in the new environment, to help rather than hinder clinical activities: *They can’t have students just walking around, … poking and looking at every little thing’ *(FG2;M1);* ‘… you want to look good, you want to be there to be able to help …’ *(FG2;M1); and* ‘… how can I be useful on this team … be accountable for something?’ *(FG2;F2).

#### Difficulty preparing for the rotation

In contrast to non-ICU clerkships, intensive care medicine is a broad specialty which students find difficult to pre-read and prepare for: *‘… you come into ICU, you might be reading up about, you know, some emergencies but you’ve no idea about what that thing is there *(FG2;M1) and:* ‘The second thing is because we none of us has really been in ICU settings before our first time in ICU, we don’t know what to expect really’ *(FG2;M1).

#### Personality of the student

Participants thought the personality of the student influenced learning outcomes, in particular how individual students coped with unfamiliarity and uncertainty. The two extremes of this were demonstrated thus: *‘… if you’re shy, … you will isolate yourself and whatever you want to do’ *(FG1;F1) and* ‘… personally that sort of thing would be just a driving force … I’d work even harder then, but that’s just me’ *(FG3;F2).

### Stage 2: Early clerkship

Transitioning to the ICU was viewed as a phase during which the new student-learner was vulnerable and reliant on others, as described by two participants: *‘If you don’t give us the means to actually find our way, … then obviously we’re going to be completely lost’ *(FG2;M2) and ‘*The students experience is kind of made or broken on the first day if somebody actually … welcomes you’ *(FG2;M1).

Four subthemes were apparent in this stage of learning. In common with the pre-clerkship period, these related to early challenges for the novice learner which influenced students’ motivation and emotional wellbeing, and their self-regulatory activities. Students had little opportunity to influence the learning environment, instead favouring support and direction from the clinical environment.

#### Initial interaction with ICU staff

A favourable initial interaction with ICU staff helps students navigate the new clerkship environment. For example: *‘Dr ____ met with us on the first morning and gave … a basic schedule so we knew what was expected … that was actually really helpful’ *(FG2;F1) and *‘this is our first formal exposure to ICU rotation, … but however, being invited to the rounds makes us a part of the team’ *(FG1;F1).

Although medical staff, in particular an ICU consultant, could help the student integrate into the new environment, ICU nurses also played a role, *‘they’d take you through things and that which is great because you don’t always see that on the wards’ *(FG1;F2).

#### Students’ sense of belonging

Students were sensitive to whether their presence in the new clerkship environment was welcomed or not, whether they were ‘*being invited to the rounds’ *(FG1;F1) or *‘allowed to be here and I’m supposed to be here’ *(FG2;F1). That this sense of belonging benefited learning was also apparent: *‘if somebody makes you feel welcomed or entitled to be there … that just breaks down all those barriers … it’s also quite motivating’ *(FG2;F1).

#### The structure/organization of the rotation

Clerkships with good structure were reported to be more beneficial for learning than unstructured ones. This was of particular importance in the unfamiliar setting of an ICU where a student was ‘a bit nervous the first day of what can I actually touch in here’ (FG2;F1). It allowed students to prioritize learning over logistical problems: *‘… you knew where you were going … you know what you needed to know … it means you can be prepared …’ *(FG3;F1).

Lack of organization led to students *‘hanging round’ *(FG3;M2) or *‘… just floating there on your own … without having a clue what’s going on’ *(FG4;M3).

#### The level of guidance available

Student guidance in ICU was viewed as either preferable or necessary. It helped overcome issues of unfamiliarity and apprehension, particularly early in the clerkship, where self-learning, in contrast to non-ICU clerkships: *‘In the ICU you can’t do that so much’ (FG2; F1); ‘… we feel so limited in what we can do, that’s why we need … some people to guide us and … tell us what we see’ *(FG2;M2)*; ‘ICU is kind of an intimidating environment … I prefer to be directed’ *(FG4;M2) *and ‘If you don’t have one of your registrars showing you around, then ICU can be a very, I guess wasteful time’ *(FG4;M3).

### Stage 3: Learning throughout the clerkship

Participants described several factors that helped or hindered their learning during the course of the clerkship. The five subthemes represented a transition from vulnerable, dependent student to an interactive, responsive learner demonstrating autonomy, self-regulatory strategies who could make the environment adapt to their learning needs.

#### Involvement in patient care

A recurring subtheme was that active involvement in patient care in the ICU was infrequent. Notwithstanding this, the value of close involvement with 1 or 2 patients – following their inpatient progress or presenting their case at daily rounds – was highlighted. It gave students a sense of inclusion in team activities, encouraged student attendance and fostered deep learning: *‘I felt like an insider, … because we were given patients’ *(FG4;M2)*; ‘… this is your patient … you need to know everything about it (sic) … it does make you focus an awful lot more and it makes you learn …’ *(FG3;F2); and *‘It was the most important moment as the medical student probably in six years, when the ICU reg called me to a real arrest’ *(FG4;M3).

#### An adjustment in learning method

As most patients were non-communicative, students were unable to practice traditional history-taking and clinical examination in the ICU. This could either promote or hinder independent learning, described as either *being ‘more of a detective than if you had someone who can talk to you … to figure out what’s going on’ *(FG2;F2) or ‘… *different to any other setting in the hospital because the patient can’t give you a history, you are very much reliant on the staff members in ICU …’ *(FG2;F1).

A further adjustment related to the volume of new information, consequent to the breadth and complexity of critical illness. Accordingly, students were challenged by the *‘information flying around … too much information in those rounds’ *(FG4;M2).

#### The value of observing and experiencing

There was a strong sense that the ICU afforded opportunities for learning infrequently experienced during other clerkships, for example: *‘… GCS was just a number in a book … before you got to see actually how Glasgow Coma Scale of three or four looks like …’ *(FG4;M3)*; ‘I had the opportunity to see people getting bad news broken to them … that was actually the only time’ *(FG3;F1)*; ‘… to actually see patients in hepatic failure or in respiratory failure on ventilation and stuff, it kind of brings it home and it makes you remember it more’ *(FG2;F1)*; ‘… you see very little in the way of emergencies unless you do ICU’ *(FG4;M1) and *‘… really good in terms of learning lines and drains and all – the invasive monitoring and stuff like that …’ *(FG2;F2).

#### Reciprocal dynamics between student and learning environment

A supportive clinical environment, such as the welcoming interaction with ICU staff, encouraged students to invest effort in the clerkship. Moreover, the non-threatening workplace, in particular the supportive consultant teachers therein, provided a safe space where students could experiment and make mistakes in the process of learning: *‘… if you’re not in an environment that will allow you to make that mistake, … you’re not going to put yourself out there …’ *(FG1;F2).

Conversely, the learning environment was sensitive to – and could change in response to – students’ behaviour. For example, regular student attendance encouraged the consultant to engage with them and set learning activities. An enthusiastic student could trigger learning activities that otherwise might not have occurred: *‘… about once a day they would give us small-group tutorials, very frequently just upon our request …’ *(FG4;M3) and *‘… if you were someone who liked to be in there and see and learn, then there were people who were willing to talk to you’ *(FG2;F2).

#### The students’ self-learning activities

Despite the perceived complexity and the need for student guidance, students could bring about their own desired learning opportunities during the rotation. These promoted learning either directly, such as when they sought out learning experiences individually or in groups, or indirectly, by bringing about a change in the responsive learning environment. For example: *‘You can always go in … if someone was on dialysis … you could go in and see that patient …’ *(FG1;F2); and* ‘… you could look at the ventilator settings, you could look at what’s around the bed … even if you weren’t directly being taught … you could actually self-teach’ *(FG3;M1).

## Discussion

Our study, apparently the first to qualitatively detail medical student ICM learning, provides a useful description of the ICU from a student perspective. While presenting challenges during preparation and induction, ICM clerkships offer students valuable learning experiences not readily available elsewhere. Factors hindering learning include the anticipation of complex illness in an unfamiliar setting, perceptions of uselessness and poor clerkship organization during student induction. Conversely, learning is facilitated by a welcoming, inclusive, supportive learning environment, where students can participate in patient-centred activities, and are motivated to pursue self-directed experiences.

The study suggests that social cognitive theory, in particular its key principles of reciprocal determinism and learner agency, offers useful insights into how students learn in the intensive care unit. Moreover, SCT can be used to explain the changing dynamics between learner and environment, as well as the factors influencing learning as students progress through different stages of an ICU clerkship. Finally, when compared with non-ICU clinical environments, the ICU may be more challenging to prepare for and more daunting to enter, but once entered, provides some unique and positive learning experiences.

We identified numerous examples of what Bandura calls the ‘complex interplay’ (p5) [24] between learner and learning environment – reciprocal determinism; the triadic relationship between the student, their behaviour and the ICU [26, 28]. Also apparent was the importance of students’ personal agency, for example effecting an educational change in the ICU environment or pursuing self-directed activities [24].

These factors, however, did not remain constant throughout the clerkship. A key theme that emerged in the data was the concept of a sequential process of familiarization, socialization, participation and affective change that developed as students engaged emotionally and physically with the clerkship. When viewed from an SCT perspective, a dynamic picture of workplace learning became apparent. Accordingly, prior to and at the start of the ICU clerkship, students were more likely to be apprehensive about the unfamiliar, complex environment and unsure about the role they would play there. Self-regulatory activities such as preparation, setting learning goals and adopting learning strategies were infrequent and students favoured high levels of support and guidance. It is likely that, at this time, the students felt ill-equipped to manipulate and influence the ICU environment and were more observers of learning than agents of learning. Consequently, the ICU environment was, in the early stages, a potential learning place awaiting a more active interaction with the student learner.

**Fig. 1:**
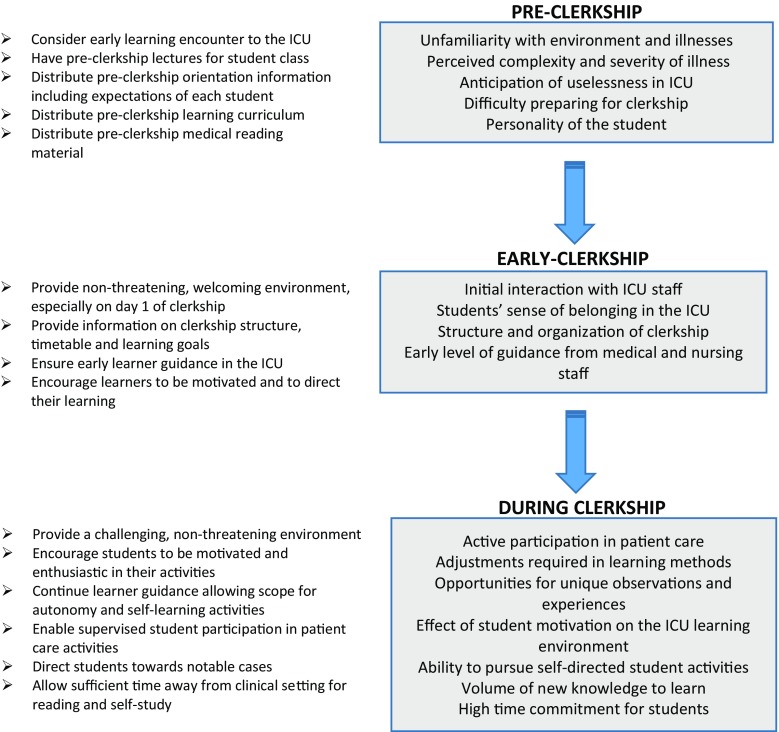
The sequential workplace model which proposes a chronological basis for student learning during a clinical clerkship (Factors influencing the three stages of the clerkship that emerged in focus group discussions are shown in the grey boxes. Some factors are in addition to those quoted in the text. Suggested interventions to optimize these factors are shown in the left-sided column)

Conversely, as the clerkship progressed, students became more confident, participatory and self-directed. This development may have coincided with the students’ greater familiarity with the ICU and its norms as well as their perceived acceptance therein. Reports of students driving and directing their learning become apparent. Students recognized their ability to influence the environment to enhance their learning. Some recognized a need to adjust their learning methods (from traditional history taking and clinical examination to a more deductive method of clinical reasoning) to pursue their learning goals. Others recognized and sought clinical experiences unique to the intensive care setting. In summary, a picture emerged of dynamic co-interaction between the active student and a mutable environment which could not be explained by social learning theory alone. These findings were combined and used to construct a clinical workplace learning model (Fig. 1). This presents a chronological basis for learning reflecting the co-dynamics between ICU and student and the factors influencing the active, cognitive learner during clinical clerkships.

Our results overlap with those of previous studies using non-sequential approaches to workplace learning. For example, Dornan et al.’s experiential model of workplace learning also emphasizes the importance for learning of active participation, supportive, encouraging healthcare staff, and effective clerkship organisation [8, 44]. The model proposed in Boor’s (2008) mixed-methods study of undergraduate obstetric clerkships, in common with our study, prioritizes learner participation, in turn influenced by environmental factors such as legitimacy and clerkship organization, and by student motivation [15]. Two previous studies [45, 46] demonstrate that the early clinical career of a medical student is notable for the challenge of induction into new clerkships, so-called ‘professional socialization’ (p113) [45] when students navigate new ‘socio-emotional space(s)’ (p364) [46], seeking an identity with attendant roles, responsibilities and expectations. The results of our study and others advocate a combination of a welcoming, ‘invitational’ (p542) [13] environment with good guidance and organization which, by supporting this period of induction [8, 10, 13, 15, 16], may promote early self-regulatory activities and learner agency. This is an important message for educators involved in providing orientation and ‘developmental space’ (p363) [46] for students.

Our study adds to existing knowledge in four main areas. First, it suggests that although student learning in the ICU and in the non-ICU setting share some overlap, ICU workplace learning is unique and may be incompletely explained by current workplace learning models. A search for models better suited to the ICU may therefore be warranted. Second, we have used our results to propose a new workplace model of ICU learning. Third, our findings, and the proposed model, are practical, suggesting ways of structuring ICU undergraduate education according to the students’ transition through the clerkship; welcoming, supportive, non-threatening and informative at the start, and delegatory, participatory and challenging as the clerkship progresses. Finally, our results apply to the ICU environment Bandura’s theory that an ‘actual learning environment’ is unique to each learner; it is a product of the ‘potential environment’ (the ICU) and the cognitive and behavioural influences of the learner therein (p196) [25]. These interdependent factors, however, are not constant. From our study, we postulate that students’ personal agency dynamically changes during a clerkship; accordingly, the ‘potential environment’ may also need to change to assist students in navigating through the stages of the clerkship and for effective learning to occur.

There are limitations to the study. With low student recruitment, data saturation was not achieved, therefore additional perspectives on ICU learning that might have emerged in further discussions may have been overlooked. Notwithstanding this, our study ‘offers new insights that contribute substantially to or challenge current understandings’ (p7), a criterion used by Malterud et al. (2015) [47] to describe a satisfactorily powered, though potentially incomplete, qualitative study. A selection bias was suggested by a predominance of positive feedback from participants. To counter this and to explore outlier views, focus group questions specifically probed for negative aspects of the clerkship. As an example of practitioner research – EOC is an ICM consultant and lecturer in the study institution – the study was at risk of bias during recruitment, data collection or during data analysis and interpretation. Third party recruitment, data triangulation, an analysis audit trail, a researcher reflexive diary and peer debriefing were used to counter this risk of bias.

## Conclusion

In summary, this study reinforces the value of the ICU as a rich, unique, social learning environment. It also suggests that effective learning of complex medical concepts even in the setting of an unfamiliar clinical workplace, is achievable, and is aided by facilitating student socialization in each new clerkship and by recognizing and responding to the sequential change in students’ interaction with the clerkship. Our proposed sequential workplace learning model may find application in the ICU setting as a tool to inform educational practice.
